# Growth inhibition and metabolomic analysis of *Xanthomonas oryzae* pv*. oryzae* treated with resveratrol

**DOI:** 10.1186/s12866-020-01803-w

**Published:** 2020-05-14

**Authors:** Huai-Zhi Luo, Ying Guan, Rui Yang, Guo-Liang Qian, Xian-Hui Yang, Jun-Song Wang, Ai-Qun Jia

**Affiliations:** 1 School of Environmental and Biological Engineering, Nanjing University of Science and Technology, Nanjing, 210094 China; 2 Engineering Research Center for Utilization of Tropical Polysaccharide Resources, Ministry of Education, Hainan University, Haikou, 570228 China; 3 Inspection and Pattern Evaluation Department, Suzhou Institute of Metrology, Suzhou, 215000 China; 4 College of Plant Protection, Nanjing Agricultural University, Nanjing, 210095 China; 5 School of Science, Hainan University, Haikou, 570228 China

**Keywords:** *Xanthomonas oryzae* pv. *Oryzae*, Resveratrol, Flagella, Structure-activity relationship, Global metabolic analysis

## Abstract

**Background:**

*Xanthomonas oryzae* pv. *oryzae* (*Xoo*) can cause destructive bacterial blight in rice. As an antibacterial, resveratrol may inhibit *Xoo* growth. This study focused on the potential structural-activity relationship of resveratrol and its derivatives against *Xoo* growth, and ^1^H-NMR-based metabolomic analysis was applied to investigate the global metabolite changes in *Xoo* after resveratrol treatment.

**Results:**

Resveratrol showed the strongest inhibitory effects on *Xoo* growth compared with its derivatives, which lacked double bonds (compounds **4**–**6**) or hydroxyls were substituted with methoxyls (compounds **7**–**9**). The IC_50_ of resveratrol against *Xoo* growth was 11.67 ± 0.58 μg/mL. Results indicated that the double bond of resveratrol contributed to its inhibitory effects on *Xoo* growth, and hydroxyls were vital for this inhibition. Interestingly, resveratrol also significantly inhibited *Xoo* flagellum growth. Based on ^1^H-NMR global metabolic analysis, a total of 30 *Xoo* metabolites were identified, the changes in the metabolic profile indicated that resveratrol could cause oxidative stress as well as disturb energy, purine, amino acid, and NAD^+^ metabolism in *Xoo*, resulting in the observed inhibitory effects on growth.

**Conclusions:**

This study showed that the double bond of resveratrol contributed to its inhibitory effects on *Xoo* growth, and hydroxyls were also the important active groups. Resveratrol could cause oxidative stress of *Xoo* cells, and disturb the metabolism of energy, purine, amino acid and NAD +, thus inhibit *Xoo* growth.

## Background

*Xanthomonas* is an important genus of gram-negative pathogenic bacteria, and can infect approximately 350 different plants [1]. *Xanthomonas oryzae* pv. *oryzae* (*Xoo*) is one of the most important bacterial pathogens in rice, and can cause destructive bacterial blight (BB) in many regions world-wide [2]. BB is an economically significant and highly devastating disease of rice, and has been reported in Asia, Africa, Australia and Latin America [1]. BB can cause at least 10% yield loss in susceptible rice varieties, and up to 60% yield loss during severe epidemics [3]. Bactericides and antibacterial agents have become indispensable tools in the control of BB, and include the most commonly used copper-based bactericides and zinc thiazole [3]. However, these bactericide agents also exhibit many shortcomings, including poor efficacy, influence on non-target organisms, high phytotoxicity, environmentally unfriendly, and bactericide resistance, so their use is relatively unsustainble [4]. Many antibiotics, such as streptomycin, have also been used to control BB [5], with benzylpenicillin, ampicillin, kanamycin, chloramphenicol, and sinobionics reported to inhibit *Xoo* growth [6]. However, over use and abuse of these antibiotic resulted in increasing bacterial resistance. Thus, development of new antibacterial agents for the control of BB is urgently required. Recently, several natural products have been reported to show antibacterial activity against *Xoo*, including peptides, graphene oxide, actinomyces, *Adathoda vasica* leaf extract, and *Datura metel* leaf extract [7].

Resveratrol, as a “model stilbene” and an important phytoalexin, is isolated from grapes, berries, peanuts, pines, and *Polygonum cuspidatum* Sieb [8]. Additionally, resveratrol can be synthesized easily with a high yield, which makes it possible to obtain large scale in low cost [9]. Resveratrol exhibits antioxidant, antiviral, anti-inflammatory, anti-fungal, and anticancer bioactivities and is a known quorum sensing inhibitor (QSI) that can inhibit *Pseudomonas aeruginosa* virulence [10]. Additionally, resveratrol can be used as a potent antibacterial to inhibit the growth of *Xanthomonas* [11] and was rescreened in the current study.

To the best of our knowledge, the main active groups of resveratrol, as well as its effects on molecular metabolic profiles and the potential inhibition mechanism against *Xanthomonas*, remain unknown. With the development of omics technologies, metabolomic has been applied to study metabolic profiles and molecular mechanisms. In this research, we investigated the potential structure-activity relationship of resveratrol and its derivatives and their inhibitory effects on *Xoo* growth. The metabolic changes in *Xoo* and underlying inhibition mechanism were also evaluated after treatment with resveratrol using ^1^H-NMR-based metabolomics [10]. Results indicated that the double bond of resveratrol contributed to its inhibition of *Xoo* growth, with hydroxyls found to be the vital active group. Furthermore, our results suggested that resveratrol could disturb energy, purine, amino acid, and NAD^+^ metabolism in *Xoo* cells, resulting in the observed inhibitory effects on growth.

## Results

### Structural identification of three Stilbenoids (1–3) and derivatives (4–9)

**Fig. 1:**
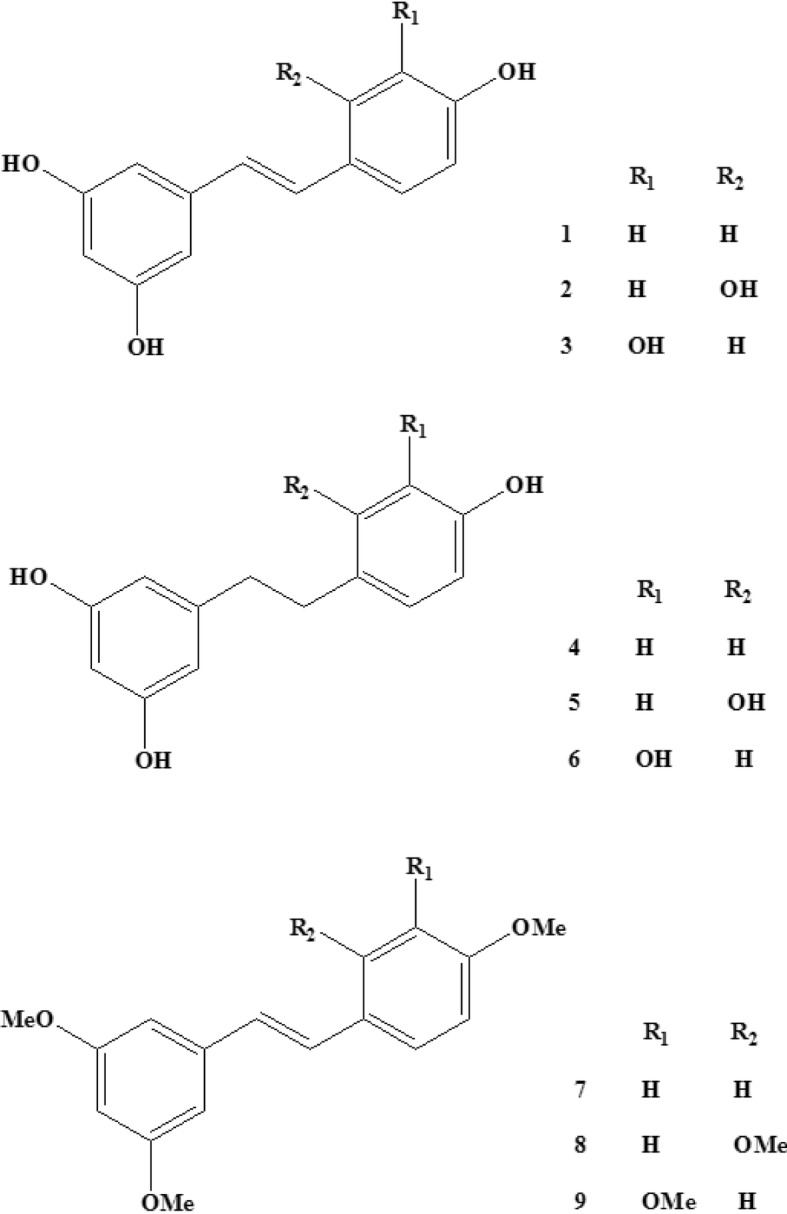
Chemical structures of compounds (**1**–**9**)

The chemical structures of three stilbenoids (**1**–**3**) and their derivatives (**4**–**9**), di-hydro-resveratrol (**4**), di-hydro-oxyresveratrol (**5**), di-hydro-piceatannol (**6**), tri-methyl-resveratrol (**7**), tetra-methyl-oxyresveratrol (**8**), and tetra-methyl-piceatannol (**9**), were shown in Fig. 1. The ^1^H- and ^13^C-NMR chemical shifts of the six derivatives (**4**–**9**) were shown in Table S1.

### Antibacterial activity of compounds (1–9) against Xoo

**Fig. 2:**
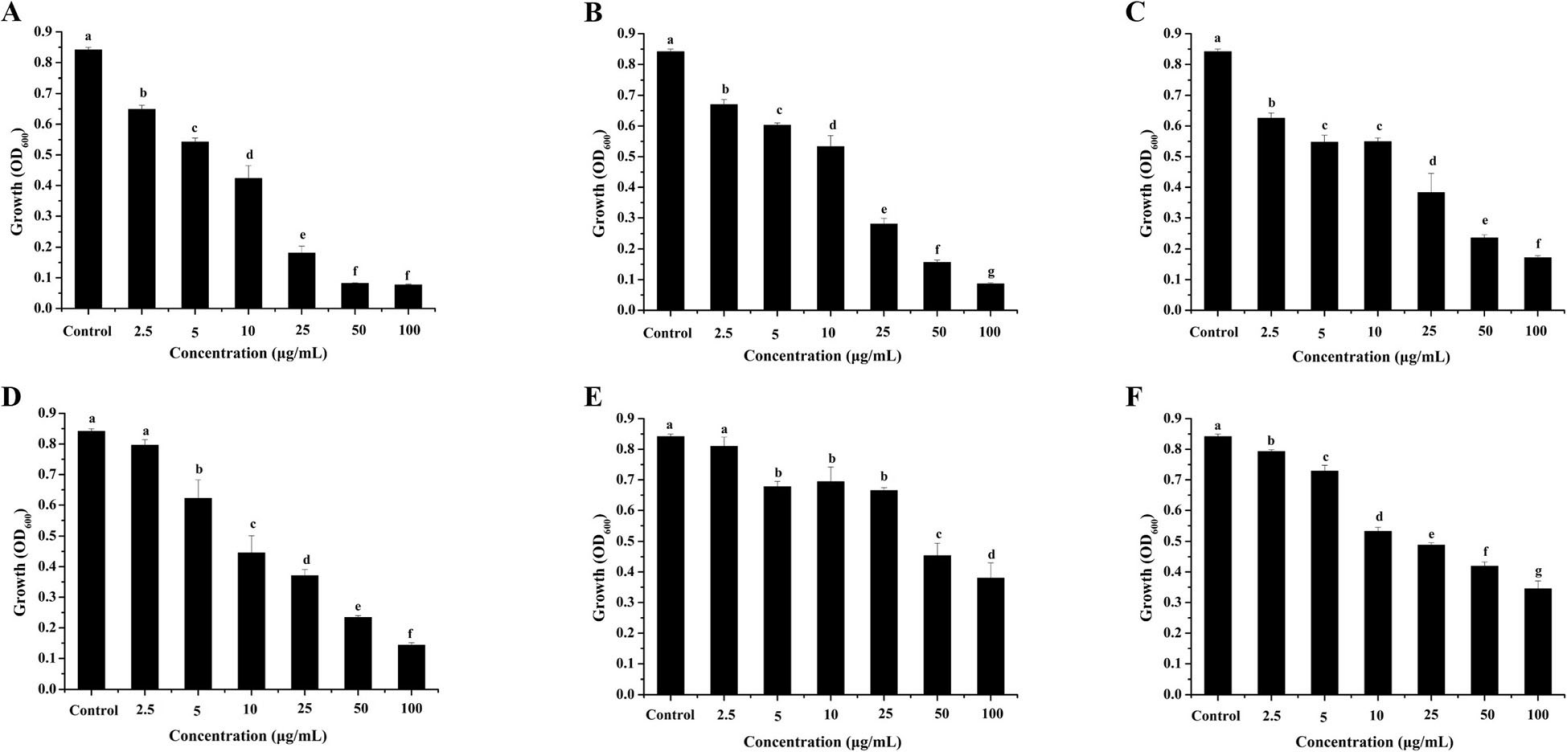
The growth of *Xoo* after treated by compounds (1–6), respectively, for 18 h, Compound (1) (**a**), Compound (2) (**b**), Compound (3) (**c**), Compound (4) (**d**), Compound (5) (**e**), and Compound (6) (**f**). Means with different lower-case letters (a, b, c, d) are significantly different (*p* < 0.05)

As shown in Fig. 2, compounds (**1**–**6**) exhibited antibacterial activity against *Xoo*, whereas compounds (**7**–**9)** had no effects on the growth of *Xoo* (data not shown). Dramatically, resveratrol (**1**) showed the strongest antibacterial activity against *Xoo* (IC_50_ 11.67 ± 0.58 μg/mL) (Table 1), and at 5 μg/mL, 25 μg/mL, and 100 μg/mL, the inhibiting percentage on *Xoo* growth was 24.66 ± 1.79, 75.84 ± 3.14, and 90.49 ± 0.28, respectively. For compounds (**2**–**6**), the inhibiting percentages on *Xoo* growth at 100 μg/mL were 89.39 ± 0.43, 78.89 ± 0.80, 82.76 ± 1.02, 54.30 ± 6.05, and 58.51 ± 3.11, respectively, and at 5 μg/mL were 16.27 ± 1.06, 23.96 ± 3.15, 25.24 ± 7.31, 18.51 ± 2.10, and 12.38 ± 2.25, respectively. As shown in Table 1, the IC_50_ values of compounds (**2–6**) on *Xoo* growth were 19.00 ± 1.00, 27.00 ± 3.61, 36.27 ± 3.75, 123.53 ± 7.66, and 115.46 ± 7.93 μg/mL, respectively.

### Effects of resveratrol on Xoo flagella

**Fig. 3:**
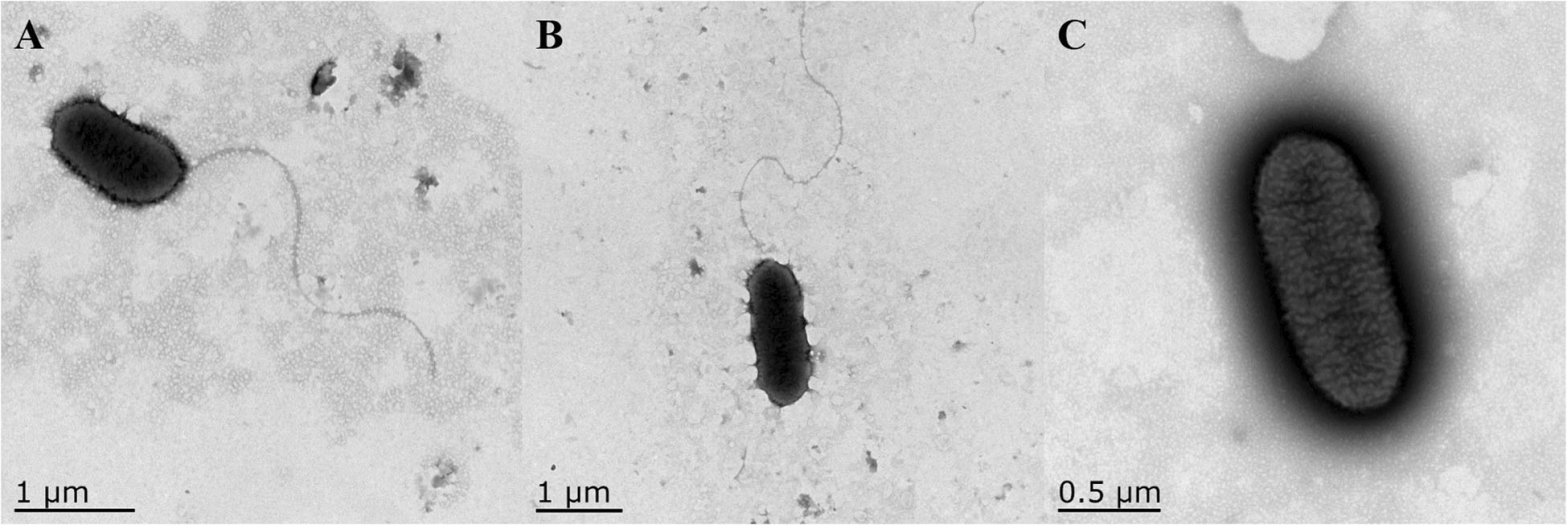
Effects of resveratrol on flagella of *Xoo*: blank control (**a**), DMSO control (**b**), and resveratrol-treated groups (**c**)

Bacterial flagella are important virulence factors for pathogenesis of animals and plants, and flagella-driven chemotaxis plays an important role in the early interaction of host plants in some plant-pathogen systems [12]. *Xoo* harbors a single polar flagellum for motility, and the function of flagella allows bacteria to move away from hazards to favorable conditions by responding to chemical signals [13]. So we investigated whether resveratrol was an inhibitor to influence the *Xoo* flagella. As shown in Fig. 3, flagella were detected on the surface of *Xoo* in the blank (Fig. 3a) and DMSO control groups (Fig. 3b), but not in the resveratrol treatment group (Fig. 3c).

### Metabolite identification and multivariate statistical analyses

**Fig. 4:**
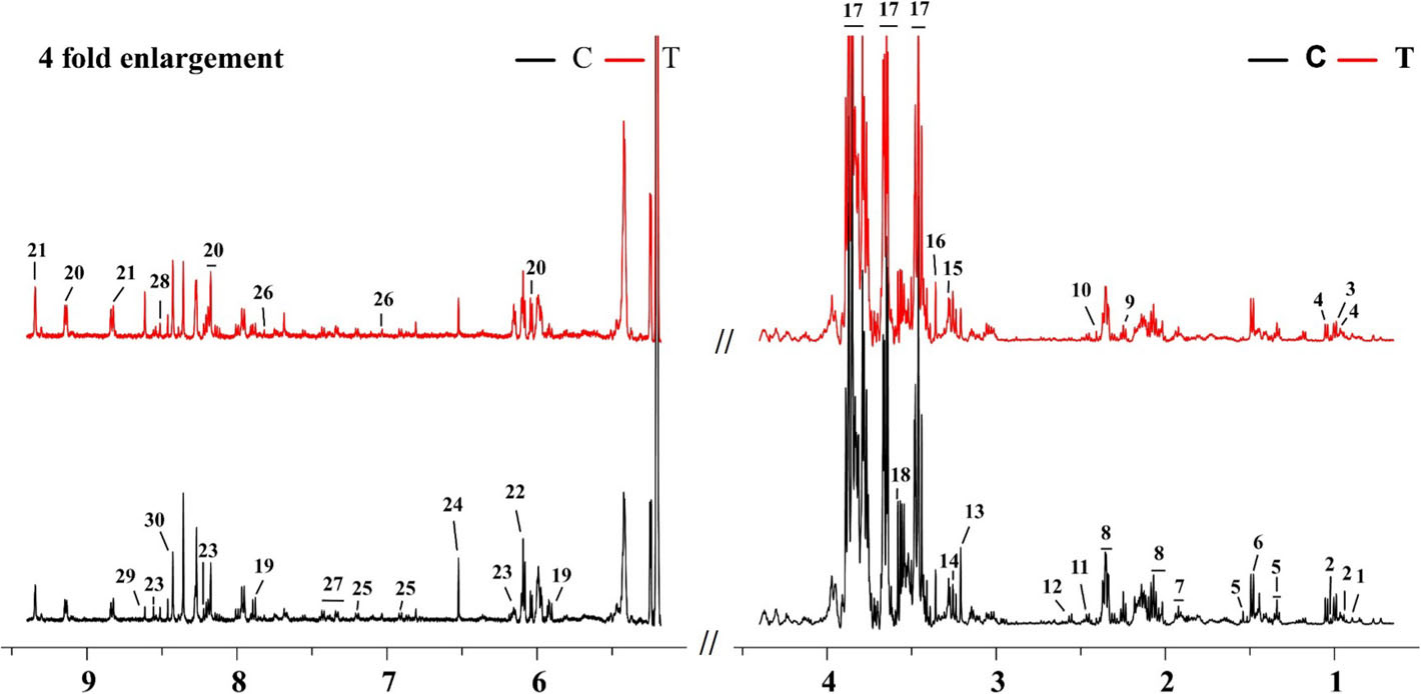
Typical 500 MHz CPMG ^1^H-NMR spectra of *Xoo* from resveratrol-treated (red line) and control groups (black line): 1, Cholate; 2, Isoleucine; 3, Leucine; 4, Valine; 5, Suberate; 6, Alanine; 7, Lysine; 8, Glutamate; 9, 2-Aminoadipate; 10, Succinate; 11, Glutamine; 12, Glutathione; 13, Choline; 14, Taurine; 15, Trimethylamine N-oxide; 16, Methanol; 17, Trehalose; 18, Glycine; 19, Uridine; 20, NAD^+^; 21, NADP^+^; 22, Inosine; 23, IMP; 24, Fumarate; 25, Tyrosine; 26, Histidine; 27, Phenylalanine; 28, ATP; 29, AMP; 30, Formate

**Fig. 5:**
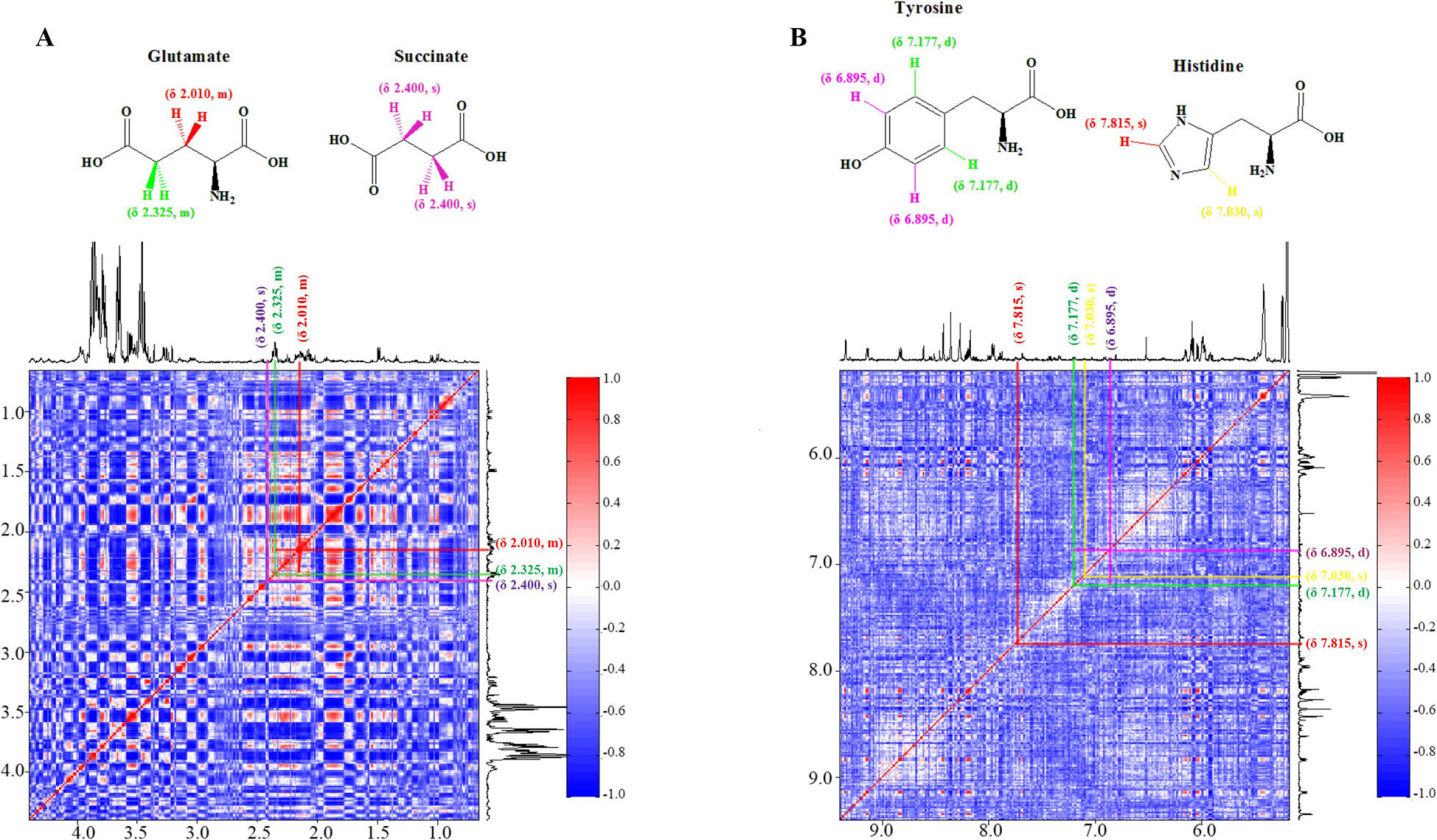
Example of two-dimensional statistical total correlation spectroscopy (STOCSY) analysis of ^1^H-NMR spectrum *Xoo* extracts to facilitate the identification of metabolites. **a** 2D STOCSY subplots from 0.65 to 4.4 ppm for the assignments of glutamate and succinate; (**b**) 2D STOCSY subplots from 5.175 to 9.4 ppm for the assignments of tyrosine and histidine

Typical 500 MHz CPMG ^1^H-NMR spectra for the resveratrol-treated (T-group) and control groups (C-group) are shown in Fig. 4, with a total of 30 metabolites assigned. Detailed information on the 30 metabolites is shown in Table 2. The STOCSY technique, which computes correlation among the intensities of all peaks in a matrix, was used for the assignment of metabolites, such as glutamate, succinate, tyrosine, and histidine (Fig. 5).

**Fig. 6:**
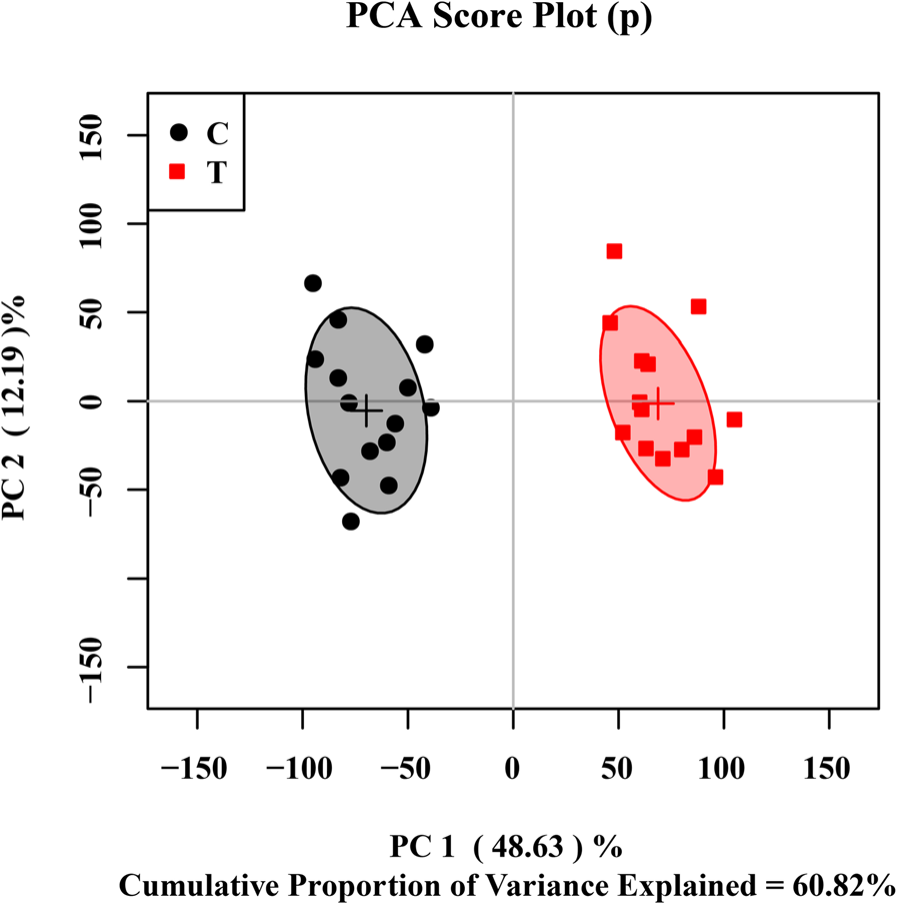
PCA score plot of ^1^H-NMR data for *Xoo*. Two PCs explained 48.63 and 12.19% of total variances in *Xoo*. Ellipses represent 95% confidence interval for each group

**Fig. 7:**
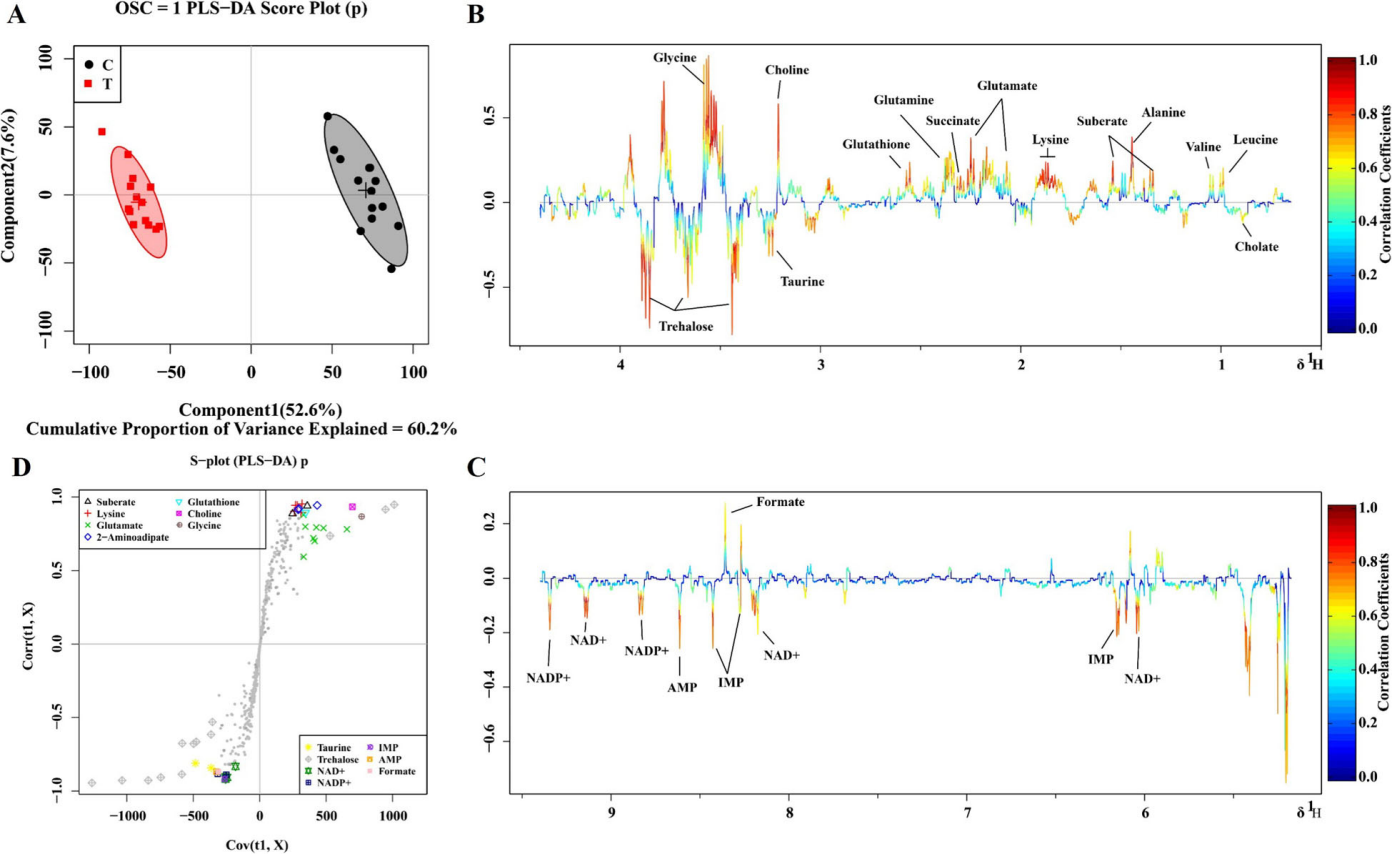
OSC-PLS-DA analysis of NMR data from *Xoo*. **a** Score plot. Component 1 and component 2 explained 60.2% of total variance in the *Xoo* sample extracts. **b, c** Color-coded loadings plots. Color bar was applied, with red and blue representing metabolites that significantly or indistinctively contributed to the separation of groups, respectively. Peaks in positive and negative status reveal decreased and increased metabolites, respectively, relative to the score plot in the resveratrol-treated group. **d** S-plot

**Fig. 8:**
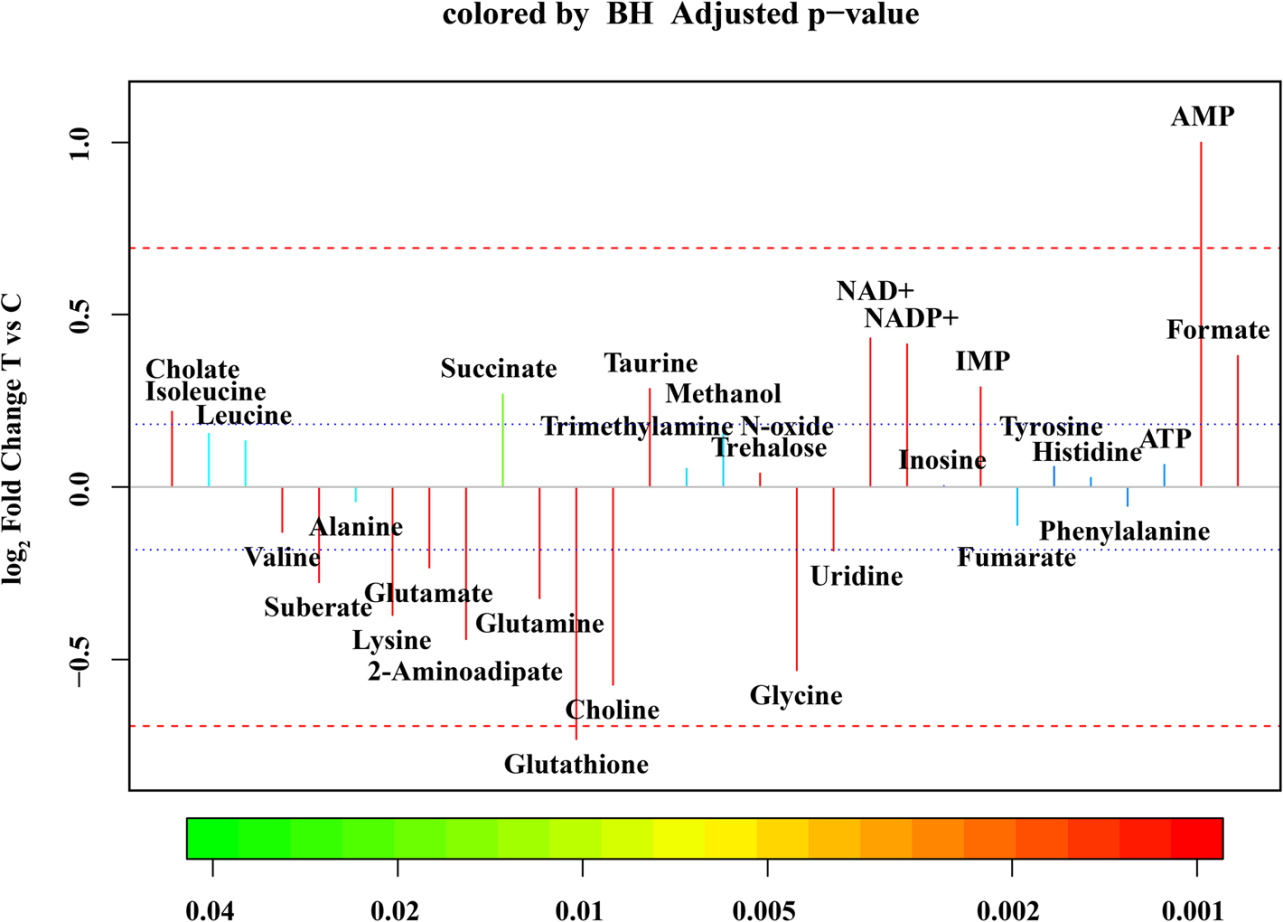
Fold-change plots color coded according to *p*-values adjusted using the Benjamini-Hochberg method, indicating the significance of differences in levels of metabolites in the resveratrol-treated and control groups

Principal component analysis (PCA) was first used to obtain an overview of variation between the C-group and T-group. Quite significant overlap was observed between the PCA score plot of two groups in Fig. 6. OSC-PLS-DA, a supervised pattern recognition technique, was used to identify the metabolic differences between the two groups. In the OSC-PLS-DA score plot (Fig. 7a), the T-group was significantly separated from the C-group. The corresponding S-plot (Fig. 7d) and the color-coded loading plots (Fig. 7b,c) were used to identify the contributions of variables between the groups. In addition, changes in metabolites were directly visualized as fold-changes in these plots, and were color-coded according to the differences of *p*-values between the groups (Fig. 8). Compared with the C-group, cholate, succinate, taurine, NAD^+^, NADP^+^, IMP, AMP, and formate significantly increased, whereas valine, suberate, lysine, glutamate, 2-aminoadipate, glutamine, glutathione, choline, glycine, uridine, and fumarate markedly decreased in the T-group. The assigned metabolites, their fold change values, and *p-*values are shown in Table 2.

## Discussion

Resveratrol, known as a natural phytoalexin, is found in some medicinal and edible plants, and shows considerable antibacterial, antioxidant, anti-inflammatory, and anticancer activities [10]. Wang reported that resveratrol can inhibit the growth of *Xanthomonas oryzae* [11]. However, the potential structure-activity relationships among resveratrol derivatives against *Xoo* growth and the influence of resveratrol on the metabolic system of *Xoo* are not known. In this study, the structure-activity relationship and the influence of resveratrol on the metabolic system of *Xoo* were investigated by ^1^H-NMR-based metabolomics. We found that: (1) The double bond between two benzene rings in resveratrol was essential to inhibit the growth of *Xoo*; (2) The hydroxyls in these benzene rings might be the main activity group involved in the inhibition of *Xoo* growth; and (3) resveratrol might cause oxidative stress, as well as disturb energy, nucleic acid, amino acid, and NAD^+^ metabolism, in *Xoo* cells.

### Inhibitory effects on growth of Xoo and the potential structure-activity relationship

Compounds (**1**–**3**) showed strong inhibition activity on the growth of *Xoo*, with resveratrol demonstrating the strongest activity among the tested compounds. Even though resveratrol exhibits antibacterial activity and can inhibit the growth of *Xanthomonas* [11, 12], the metabolomic mechanisms of the inhibition are still unknown. It was reported that the mechanisms were complex and different for different inhibitors. Difficidin and bacilysin could influence the protein and cell wall synthesis and cell division to inhibit the growth of *Xanthomonas* [14]. Phenazine-1-carboxylic acid influenced the energy metabolism and disturbed the redox balance in *Xoo* [15, 16]. So as a famous antioxidant, resveratrol maybe had effects on the energy metabolism and redox balance of *Xoo*. Subsequently, we investigated the structure-activity relationships among resveratrol and its derivatives. Compared with compounds (**1**–**3**), compounds (**4**–**6**), which lacked the double bond, showed weaker inhibitory effects on the growth of *Xoo*. Thus, the double bond in the skeleton of compounds (**1**–**3**) likely played an important role in the inhibition of *Xoo* growth. Compared with compounds (**1**–**3**), all hydroxyls were substituted by methoxyls on the benzene rings in compounds (**7**–**9**). Interestingly, compounds (**7**–**9**) had no inhibitory effects on *Xoo* growth, indicating that the hydroxyls were the vital active group for the inhibitory effects on *Xoo* growth observed for compounds (**1**–**3**). For many inhibitors, the double bond and the hydroxyls played important roles in the anti-*Xoo* activity, such as sphaeropsidin A, the main phytotoxin produced by *Diplodia cupressi* [17], and it was reported that stilbenoids with at least one free hydroxyl group as a common structural feature suggested an association with the antimicrobial activity, such as against *Bacillus subtilis* and *Pseudomonas syringae* [18]. In addition, the literature also showed that the hydroxyls and double bond of stilbenoids were the vital active group for QSI activity, resveratrol showed remarkable QSI activity to *C. violaceum* and *P. aeruginosa* [8], and antibacterial activity against *S. aureus*, *E. coli* O157, *S. typhimurium*, and *V. parahemolyticus*, but these bioactivities were vanished after the hydroxyl group at position 3 was substituted by a glucosyl group [19, 20]. Thus, the hydroxyls in these benzene rings and the double bond between two benzene rings in resveratrol were vital for antibacterial activity.

### Effects of resveratrol on the flagella of Xoo

Flagella are essential for the virulence of organisms such as *Pseudomonas* and *Xanthomonas*, and play an important role in the pathogenesis of other mucosal infections as they are directly related to chemotaxis and motility [21]. Comparison of the control and resveratrol-treated groups (Fig. 3) indicated that resveratrol significantly inhibited flagellum growth of *Xoo*. This lack of flagella could caused efficiency of chemotaxis and motility, resulting in a decrease in virulence and pathogenicity [21, 22].

### Oxidative stress

Oxidative stress reflects an imbalance between the generation of reactive oxygen species (ROS) and antioxidant defense, with antioxidative enzymes playing a central role in determining individual risk of developing oxidative stress. The glutathione (GSH) redox system is an important antioxidant defense mechanism, in which glutathione peroxidase (GPx) and glutathione reductase (GR) catalyze the interconversion between reduced glutathione (GSH) and glutathione disulfide (GSSG) [10]. The level of GSH decrease indicated that it was excessive consumption to counteract oxidative stress because of its natural antioxidant in the cells. Additionally, GSH can clear electrophilic exogenous substances and influence detoxification under catalyzing by glutathione S-transferase (GST), which is an adaptive mechanism to reduce toxic effects. After *Xoo* was treated with resveratrol, the detoxification mechanism may have exacerbated the depletion of GSH, resulting in accelerated synthesis and significantly decreased levels of precursors such as glutamate and glutamine [23].

Cell membranes are susceptible to oxidative damage due to unsaturated fatty acids [24]. Choline and phosphocholine are crucial for the structural integrity of cell membranes. In the present study, choline was remarkably decreased in the resveratrol-treated group, indicating that choline was excessively consumed to repair damaged membranes caused by ROS. Because of the strong antioxidant activity and protective effects, taurine showed protective effects against oxidative stress [25]. The increase in taurine indicated its requirement was improving to protect the cell from oxidative stress after treatment with resveratrol [26, 27]. Thus, the decrease in choline and increase in taurine indicated that *Xoo* underwent severe oxidative damage after treatment with resveratrol.

### Energy metabolism

Compared with the control group, the level of succinate was significantly increased, whereas the level of fumarate was remarkably decreased after *Xoo* was treated with resveratrol. Both succinate and fumarate are intermediates of the tricarboxylic acid (TCA) cycle, and succinate can be converted to fumarate with succinate dehydrogenase [10]. In normal conditions, most energy is produced through the TCA cycle under aerobic respiration [26]. The increase in succinate and decrease in fumarate indicated that the TCA cycle was disrupted and energy metabolism was disturbed, which, in turn, inhibited growth of *Xoo*.

In addition, adenosine monophosphate (AMP) showed a remarkable increase in the resveratrol-treated group. AMP-activated protein kinase (AMPK) can regulate multiple biological processes regarding cell growth, especially cellular energy homeostasis [28]. Therefore, the change in AMP level might disturb the AMPK pathway and influence the energy metabolism balance.

### Purine metabolism

Inosine, used for nucleoside synthesis with inosine monophosphate (IMP), adenosine monophosphate (AMP), and guanosine monophosphate (GMP), provides substrates for the enzymatic biosynthesis of DNA and RNA [23]. The inosine level did not significantly increase, whereas the levels of IMP and AMP did show remarkable increase. ATP hydrolyzes into ADP or AMP to produce energy for the cell, and IMP can convert into AMP by adenylosuccinate lyase [29]. Interestingly, the ATP level did not significantly increase, so the AMP/ATP ratio increased after treatment with resveratrol, indicating that the balance between the production and consumption of ATP was disturbed [30].

### Amino acids metabolism

Valine showed a remarkable decrease in the resveratrol-treated group. Valine is a branched-chain amino acid (BCAA, including leucine, isoleucine, and valine) [31]. BCAAs are essential amino acids in vivo and act as vital substrates to regulate protein synthesis [10]. The decrease in valine observed in the present study suggests that the normal protein synthesis of *Xoo* broke-down due to resveratrol treatment. As proteins are vital substrates for organisms, the growth of *Xoo* was inhibited after exposure to resveratrol. In addition, some evidence indicates that BCAAs are also nutrient signals that regulate many cellular functions, including cell growth, protein transcription, autophagy, and proliferation [31–33].

Compared with the control group, the level of lysine was significantly decreased in the resveratrol-treated group. Lysine is an essential amino acid and cannot be synthesized with in the cell. Lysine plays a crucial role in the production of carnitine, which can facilitate the oxidization of fatty acids into acetyl CoA, which then enters the TCA cycle. The decrease in lysine in the resveratrol-treated group suggests a facilitated conversion to carnitine, which led to the decrease of growing in extreme condition [26].

### NAD^+^ metabolism

The levels of NAD^+^ and NADP^+^ were significantly increased in the resveratrol-treated group, indicating that the metabolism of NAD^+^ was disturbed. NAD^+^ was either de novo synthesized from tryptophan or through the niacin salvage pathway. For the niacin pathway, nicotinamide (NAM) was converted to nicotinamide ribotide (NMN) by nicotinamide phosphoribosyl transferase (Nampt), then NMN was converted to NAD^+^ by the catalysis of nicotinamide mononucleotide adenylyl transferase (Nmnat) [34]. The significant increase in NAD^+^ observed in the current study indicated that resveratrol enhanced the activity of Nmnat. Through increasing levels of NAD^+^, Nampt-mediated NAD^+^ biosynthesis could influence metabolic responses, stress resistance, and cellular differentiation in different cell types [35], and thereby regulate the activity of NAD^+^-consuming enzymes to improve cellular resistance to damage and stress, and enhance the ability of cells to survive stressful conditions [36]. In conclusion, the increase in NAD^+^ reflects a self-repair mechanism of cells to counteract resveratrol-induced damage.

## Conclusion

Resveratrol showed the strongest inhibition on the growth of *Xoo* among the nine resveratrol derivatives examined. The potential structure-activity relationship indicated that the double bond of resveratrol contributed to its inhibitory effects on *Xoo* growth, and the hydroxyls were the vital active group for the inhibitory effects. In addition, resveratrol significantly inhibited *Xoo* flagella, which might cause chemotaxis and motility deficiency, and thus a decrease in virulence and pathogenicity. The ^1^H NMR-based metabolomics approach was applied to study the mechanism of resveratrol against *Xoo* growth. A total of 30 metabolites were identified and assigned. Multivariate statistical analysis highlighted the altered metabolites and indicated that resveratrol could cause oxidative stress and disturb energy, purine, amino acid, and NAD^+^ metabolism in *Xoo* cells, thus inhibiting *Xoo* growth. The ^1^H-NMR-based metabolomics approach is a rapid and convenient tool for investigating the mechanism of resveratrol against *Xoo* growth.

## Methods

### Bacterial strains and culture conditions

The *Xanthomonas oryzae* pv. *oryzae* (*Xoo*) strain PXO99^A^ was kindly provided by Prof. G. L. Qian (Nanjing Agricultural University) [37]. *Xoo* was cultivated at 28 °C on nutrient agar (NA) medium in plates or in nutrient broth (NB) medium in flasks. NA medium consists of 10 g of sucrose, 5 g of peptone, 3 g of beef extract, 1 g of yeast powder, and 15 g of agar powder per liter of distilled water. NB medium contained the same components but lacked agar powder.

### Chemicals

Resveratrol (**1**) was isolated from *Smilax china* (purity > 95%). Oxyresveratrol (**2**) and piceatannol (**3**) were purchased from Hangzhou Great Forest Biomedical Ltd. (purity > 95%) (Zhejiang, China). Six derivatives of the above three stilbenoids (**1–3**), (di-hydro-resveratrol (**4**), di-hydro-oxyresveratrol (**5**), di-hydro-piceatannol (**6**), tri-methyl-resveratrol (**7**), tetra-methyl-oxyresveratrol (**8**), and tetra-methyl-piceatannol (**9**)) were synthesized in our lab. All other chemicals used in this study were purchased from Sigma Chemical (St. Louis, MO, USA).

### Minimum inhibitory concentrations (MIC)

MICs of compounds (**1**–**9**) were determined by following the two-fold dilution method [38]. In brief, overnight cultures of *Xoo* (1%, v/v) were resuspended in fresh nutrient agar (NB) medium in the presence of the samples (0.001–0.1 mg/mL for compounds (**1**–**6**) and 0.01–1 mg/mL for compounds (**7**–**9**)) in 96-well plates, then incubated at 28 °C and 180 rpm for 18 h. The MICs of compounds (**1**–**9**) against *Xoo* are shown in Table 3.

**Table 1:** Half maximal inhibitory concentrations (IC_50_) of compounds **1**–**9** on the growth of *Xoo*

Compounds	IC_50_ (μg/mL)
1	11.67 ± 0.58 c
2	19.00 ± 1.00 c
3	27.00 ± 3.61 c
4	36.27 ± 3.75 b
5	123.53 ± 7.66 a
6	115.46 ± 7.93 a
7	nd
8	nd
9	nd

*nd* not detected

IC_50_ values were obtained by interpolation from linear regression analysis. Values are presented as mean ± SD (*n* = 3), and means in the same column with different lower case letters (a, b, c) are significantly different (*p* < 0.05)

**Table 2:**
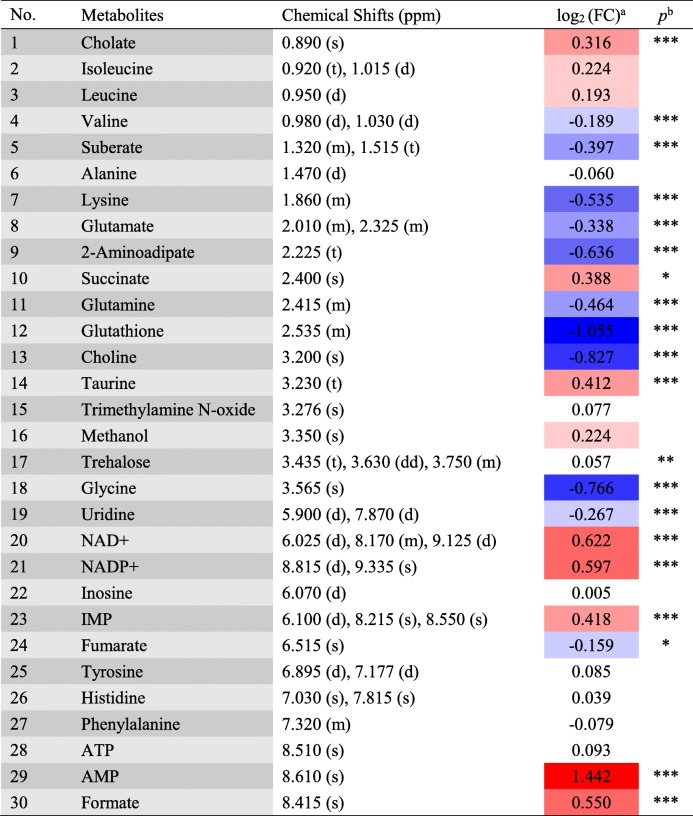
Important Metabolites Assignments in *Xoo*, Their Fold Change Values, and Associated *p* Values

a Color coded according to the fold change value, red represents increased and blue represents decreased concentrations of metabolites

b *p*-values corrected by BH (Benjamini Hochberg) methods were calculated based on a parametric Student’s *t*-test or a nonparametric Mann-Whitney test (dependent on the conformity to normal distribution). *p < 0.05, **p < 0.01, ***p < 0.001

**Table 3:** Minimum inhibitory concentrations (MICs) of the compounds 1–9 against Xoo

Compounds	MIC (μg/mL)
1	1.56
2	1.56
3	6.25
4	3.13
5	6.25
6	6.25
7	> 1000
8	> 1000
9	> 1000

### Antibacterial investigation of compounds (1–9) against Xoo

The antibacterial activity of compounds (**1**–**9**) against *Xoo* was investigated as per previous research, with some modifications [39]. Briefly, overnight cultures of *Xoo* (1%, v/v) were resuspended in the fresh NB medium supplemented with samples at concentration gradients in test tubes, then incubated at 28 °C and 180 rpm for 18 h. The concentrations of compounds (**1**–**6**) were 0, 2.5, 5, 10, 25, 50, and 100 μg/mL, and compounds (**7**–**9**) were 0, 50, 100, 200, 500, and 1000 μg/mL. The same amount of DMSO was used as the control. Then cultures were analyzed for antibacterial activity at 600 nm by a spectrophotometer (BioTek, Vermont, USA). Percentage inhibition was calculated as follows:

$$ \mathrm{Inhibition}\%=\left[1-\left({\mathrm{A}}_{\mathrm{i}}/{\mathrm{A}}_0\right)\right]\times 100 $$

where A_i_ is the OD_600_ of the cultures with compounds (**1–9**) and A_0_ is the OD_600_ of the control culture.

### Bacterial growth measurement

The effects of resveratrol on the *Xoo* growth were measured by following the previous methods, with some modifications [40]. Briefly, overnight culture of *Xoo* (1%, v/v) were resuspended in the fresh NB medium supplemented with resveratrol at different concentrations (0, 2.5, 5, 10, 25, 50, and 100 μg/mL), then incubated at 28 °C and 180 rpm. The same amount of DMSO was used as the control. The OD_600_ values of the culture were measured every 2 h for up to 24 h by a microplate reader (BioTek, Vermont, USA). The *Xoo* growth was evaluated by plotting the values of OD_600_ against time. The results were shown in Fig. S1.

### Transmission electron microscope of the Xoo flagella

*Xoo* flagella were detected by transmission electron microscopy (TEM) following the methods, with some modifications [22]. Each *Xoo* overnight culture was diluted into fresh NB medium at 1% (v/v), then incubated at 28 °C and 180 rpm for 18 h. Resveratrol was added at 11.67 μg/mL to ensure 50% effect on growth. The suspension was deposited onto grids, then stained with 2% uranyl acetate for 30 s and dried for 10 min at room temperature, with the flagella then observed by TEM (Tecnai 12, Philips, Holland).

### Extraction of Xoo metabolite

Metabolites of *Xoo* were extracted according to our previous study, with some modifications [10]. Each *Xoo* overnight culture was diluted into 30 mL of fresh NB medium at 1% (v/v) in Erlenmeyer flasks, then incubated at 28 °C and 180 rpm for 18 h (OD_600_ ≈ 0.82). Resveratrol was added at 11.67 μg/mL to ensure 50% effect on growth (OD_600_ ≈ 0.41). To ensure bacterial equality, two Erlenmeyer flasks of culture were combined to obtain one treated group sample. The same amount of DMSO was used in the control group. Fourteen biological replicates were used for the treatment and control groups, respectively. After incubation, the cell culture was chilled by brief incubation on ice. The cell pellet was obtained by centrifugation at 12,000 rpm for 15 min at 4 °C. Subsequently, the cell pellet was washed three times with phosphate-buffered saline (PBS), and then transferred to a 10-mL microtube equipped with 3.8 mL of precooled methanol/water (1/0.9, v/v), and stored at 4 °C until use. Mixtures were then extracted with a homogenizer for 5 min on ice, with 2 mL of chloroform added. After vortexing, place the mixtures on ice for 10 min, then centrifuged at 12,000 rpm for 15 min at 4 °C. Subsequently, the supernatants were transferred to new centrifugal tubes and treated under vacuum with a Speed-Vac Concentrator (Thermo SAVANT, SC110A-230) to completely remove methanol. The supernatants were stored at − 80 °C overnight, and then lyophilized in a freeze drier. All samples were stored at − 80 °C for further analysis.

### Nuclear magnetic resonance (NMR) measurements

According to published methods [10], the lyophilized extracts were dissolved in 600 μL of 99.8% D_2_O PBS buffer (pH 7.4) equipped with 0.05% (w/v) sodium 3-(trimethylsilyl) propionate-2,2,3,3-*d*_4_ (TSP). After vortexing, the mixtures were centrifuged at 12000 rpm for 15 min to discard sediments. The supernatants were transferred to new NMR tubes for ^1^H-NMR analysis.

The ^1^H-NMR spectra of samples were recorded on a Bruker AVANCE III 500 MHz NMR spectrometer at 298 K. D_2_O was used for field frequency locking, TSP was used as the chemical shift reference (^1^H, 0.00 ppm). A transverse relaxation-edited Carr-Purcell-Meiboom-Gill (CPMG) sequence [90(τ-180-τ) nacquisition] with a total spin-echo delay (2 nτ) of 40 ms was used to suppress the signals of proteins. ^1^H-NMR spectra were measured with 128 scans in 32 K data points with a spectral width of 10, 000 Hz. The spectra were Fourier transformed after multiplying the free induction decays (FIDs) by an exponential weighting function corresponding to a line-broadening of 0.5 Hz.

### Data preprocessing and peak assignments

Before analysis, the ^1^H-NMR spectrum was manually phased and baseline corrected using Bruker Topspin 3.0 software (Bruker GmbH, Karlsruhe, Germany) and referenced to TSP at 0.0 ppm. Subsequently, the ASCII format files were obtained by the convert of MestReNova (Version 8.0.1, Mestrelab Research SL). And then these files were read into R software (http://cran.rproject.org) for multivariate analysis. The spectra between 0.2 and 10 ppm were segmented with an average binning of 0.005 ppm. The regions influenced by the residual resonance of water was cut off between 4.4 and 5.175 ppm. Then, all spectra were conducted probabilistic quotient normalization and mean-centered before multivariate statistical analysis.

The NMR resonances were assigned by querying metabolomics databases, including the Human Metabolome Database (HMDB, http://www.hmdb.ca) and Madison-Qingdao Metabolomics Consortium Database (MMCD, http://mmcd.nmrfam.wisc.edu), in conjunction with the Chenomx NMR suite 7.5 (Chenomx Inc., Edmonton, Canada) and statistical total correlation spectroscopy (STOCSY) [10].

### Multivariate data analysis

Multivariate statistical analysis was applied to the NMR data, and included principal component analysis (PCA) and supervised orthogonal signal correction partial least-squares discriminant analysis (OSC-PLS-DA). Unsupervised PCA was first used to reduce the dimensionality of the imported NMR data, and some new latent variables, principal components, were obtained, with such components smaller than variables before transformation. And then filter out irrelevant effects and maximize the discrimination of inter group differences by supervised OSC-PLS-DA. The OSC was applied prior to PLS-DA to filter out unrelated variables not concerning class discrimination to minimize the influence of unrelated signals.

The quality of the OSC-PLS-DA model was evaluated by repeated two-fold cross-validation. The R^2^ and Q^2^ parameters reflected the prediction ability and the goodness-of-fit of the constructed models. In order to the further validate the supervised model, a permutation test (2000 times) was performed [41]. And color-coded loading plots were constructed to reveal variables that contributed to group separation. The fold-change values of metabolites and their associated *p*-values corrected by the Benjamini and Hochberg-adjusted method were calculated and visualized in colored tables [42]. In addition, receiver operating characteristic (ROC) curves were used to verify the classifier performance of the established OSC-PLS-DA models after 200 times repeated two-fold cross-validation [10].

### Univariate statistical analysis

Univariate analyses, including nonparametric Mann-Whitney tests and the parametric Student’s *t-*tests [10], which were used to detect difference in crucial metabolites between groups. The fold-change values of the identified metabolites as well as *p*-values between groups were calculated. Then false discovery rate was controlled by adjusting p-values according to the Benjamini-Hochberg method when proceeding with multiple comparisons.

### Statistical analysis

All experiments were run in triplicate, and experimental results were expressed as means ± standard deviation or averages. Data were analyzed by one-way analysis of variance (ANOVA) and Duncan’s multiple range test were performed using SPSS version 17.0 (SPSS Inc., Chicago, IL, USA) statistical software with a significant *P* value of *p* < 0.05.

## Supplementary information

**Additional file 1: Table S1.**^13^C-NMR (125 MHz) spectroscopic data of compounds (4–9). **Figure S1.** Effects of resveratrol on *Xoo* growth with different concentrations.

## Data Availability

All data generated or analysed of this study are described in this paper.

## Abbreviations

Xoo: Xanthomonas oryzae pv. Oryzae
BB: Bacterial blight
QSI: Quorum sensing inhibitor
PCA: Principal component analysis
MIC: Minimum Inhibitory Concentrations
ROS: Reactive oxygen species
GSH: Glutathione
GPx: Glutathione peroxidase
GR: Glutathione reductase
GST: Glutathione S-transferase
TCA: Tricarboxylic acid
AMP: Adenosine monophosphate
AMPK: AMP-activated protein kinase
IMP: Inosine monophosphate
GMP: Guanosine monophosphate
BCAA: Branched-chain amino acid
NAM: Nicotinamide
NMN: Nicotinamide ribotide
Nampt: Nicotinamide phosphoribosyltransferase
Nmnat: Nicotinamide mononucleotide adenylyltransferase
CPMG: Carr-Purcell-Meiboom-Gill
FIDs: Free induction decays
MMCD: Madison-Qingdao Metabolomics Consortium Database
OSC-PLS-DA: Orthogonal signal correction partial least-squares discriminant analysis
ROC: Receiver operating characteristic
ANOVA: One-way analysis of variance
NMR: Nuclear Magnetic Resonance
