# Supplementary material for: Growth inhibition and metabolomic analysis of Xanthomonas oryzae pv. oryzae treated with resveratrol
Source: BMC Microbiol. 2020 May 14;20:117. doi: 10.1186/s12866-020-01803-w (PMC7227335; doi:10.1186/s12866-020-01803-w)
Supplement: Supplementary file 1 — Additional file 1: Table S1.13C-NMR (125 MHz) spectroscopic data of compounds (4–9). Figure S1. Effects of resveratrol on Xoo growth with different concentrations. [file 12866_2020_1803_MOESM1_ESM.docx]

**Journal:** BMC Microbiology

Supplementary Material

**Resveratrol inhibits the growth of** ***Xanthomonas oryzae* pv*. oryzae* and the** **metabolomic analysis**

Huai-Zhi Luo^a,b,1^, Ying Guan^c,1^, Rui Yang^a,1^, Guo-Liang Qian^d^, Xian-Hui Yang^e,^*, Jun-Song Wang^a,^*, and Ai-Qun Jia^a,b,^*

Affiliations

^a^ School of Environmental and Biological Engineering, Nanjing University of Science and Technology, Nanjing 210094, China;

^b^Engineering Research Center for Utilization of Tropical Polysaccharide Resources, Ministry of Education, Hainan University, Haikou 570228, China;

^c^ Inspection and Pattern Evaluation Department, Suzhou Institute of Metrology, Suzhou 215000, China;

^d^ College of Plant Protection, Nanjing Agricultural University, Nanjing 210095, China;

^e^ School of Science, Hainan University, Haikou 570228, China.

* **Corresponding author.**

*E-mail address:* [aiqunj302@njust.edu.cn](mailto:aiqunj302@njust.edu.cn) (A-Q. Jia)

^1^These authors contributed equally to this work.

1. **The synthesis procedures of 6 derivatives (di-hydro-resveratrol (4), di-hydro-oxyresveratrol (5), di-hydro-piceatannol (6), tri-methyl-resveratrol (7),** [**tetra-methyl**](javascript:void(0);)**-oxyresveratrol (8), and** [**tetra-methyl**](javascript:void(0);)**-piceatannol (9))**
   1. Synthesis of Di-hydro Derivatives of Resveratrol, Oxyresveratrol and Piceatannol

Di-hydro derivatives of resveratrol (**1**), oxyresveratrol (**2**) and piceatannol (**3**) were obtained by published methods with some modifications [1]. Briefly, 100 mg compounds (**1**-**3**) were dissolved in 50 mL ethyl alcohol, respectively, then 15 mg 10% Pd/C was added as the catalyst. Subsequently, the mixtures were stirred under H_2_ for 6 h at room temperature. After react completely, the reaction mixtures were filtered to remove the catalyst, and filter liquor was evaporated to dryness to get the di-hydro-resveratrol (**4**) (102 mg), di-hydro-oxyresveratrol (**5**) (101 mg), and di-hydro-piceatannol (**6**) (96 mg), respectively.

- 1. Synthesis of Tri-methyl-resveratrol, [Tetra-methyl](javascript:void(0);)-oxyresveratrol and [Tetra-methyl](javascript:void(0);)-piceatannol

Tri-methyl-resveratrol (**7**), [tetra-methyl](javascript:void(0);)-oxyresveratrol (**8**), and [tetra-methyl](javascript:void(0);)-piceatannol (**9**) were obtained by the published method with some modifications [2]. Briefly, 100 mg compounds (**1**-**3**) were dissolved in 20 mL acetone, respectively. Then K_2_CO_3_ (340 mg, 6 eq) and CH_3_I (0.15 mL, 6 eq) were added into the solutions, respectively. The reaction mixtures were refluxed for 24 h at room temperature. Subsequently, the obtained residues were purified by silica gel column chromatography (CH_2_Cl_2_) to yielded tri-methyl-resveratrol (**7**) (45 mg), [tetra-methyl](javascript:void(0);)-oxyresveratrol (**8**) (44 mg) and [tetra-methyl](javascript:void(0);)-piceatannol (**9**) (27 mg).

1. **Structural identification of 3 stilbenoids (1-3) derivatives (di-hydro-resveratrol (4), di-hydro-oxyresveratrol (5), di-hydro-piceatannol (6), tri-methyl-resveratrol (7),** [**tetra-methyl**](javascript:void(0);)**-oxyresveratrol (8), and** [**tetra-methyl**](javascript:void(0);)**-piceatannol (9))**

Compound **4**, Dihydroresveratrol. ^1^H-NMR (500 MHz, CD_3_OD): 6.96 (2H, d, *J* = 8.2 Hz, H-2’, 6’), 6.68 (2H, d, *J* = 8.2 Hz, H-3’, 5’), 6.13 (3H, m, H-2, 4, 6), 2.74 (4H, m, H-7, 8) [3].

Compound **5**, Dihydroxyresveratrol. ^1^H-NMR (500 MHz, CD_3_OD): 6.79 (1H, d, *J* = 8.1 Hz, H-6’), 6.29 (1H, d, *J* = 8.1 Hz, H-5’), 6.20 (3H, m, H-2, 6, 3’), 6.09 (1H, s, H-4), 2.71 (4H, m, H-7, 8) [1].

Compound **6**, Dihydropiceatannol. ^1^H-NMR (500 MHz, CD_3_OD): 6.66 (1H, d, *J* = 8.0 Hz, H-5’), 6.61 (1H, d, *J* = 1.5 Hz, H-2’), 6.48 (1H, dd, *J* = 8.0, 1.5 Hz, H-6’), 6.14 (2H, d, *J* = 1.8 Hz, H-2, 6), 6.11 (1H, m, H-4), 2.69 (4H, m, H-7, 8) [1].

Compound **7**, Tri-methyl-resveratrol. ^1^H-NMR (500 MHz, CDCl_3_): 7.49 (2H, d, *J* = 8.7 Hz, H-2’, 6’),7.10 (1H, d, *J* = 16.3 Hz, H-8), 6.94 (2H, d, *J* = 8.7 Hz, H-3’, 5’), 6.96 (1H, d, *J* = 16.3 Hz, H-7), 6.70 (2H, d, *J* = 2.3 Hz, H-2, 6), 6.43 (1H, t, *J* = 4.5, 2.3 Hz, H-4), 3.86 (3H, s, H_MeO_-4’), 3.86 (6H, s, H_MeO_-3, 5) [4].

Compound **8**, 1-[(1*E*)-2-(3,5-Di-methoxyphenyl)ethenyl]-2,4-dimethoxybenzene. ^1^H-NMR (500 MHz, CDCl_3_): 7.54 (1H, d, *J* = 8.6 Hz, H-6’),7.43 (1H, d, *J* = 16.4 Hz, H-8), 7.00 (1H, d, *J* = 16.4 Hz, H-7), 6.72 (2H, d, *J* = 2.3 Hz, H-2, 6), 6.56 (1H, dd, *J* = 8.5, 2.4 Hz, H-5’), 6.51 (1H, d, *J* = 2.4 Hz, H-3’), 6.41 (1H, t, *J* = 4.5, 2.3 Hz, H-4), 3.90 (3H, s, H_MeO_-2’), 3.86 (9H, s, H_MeO_-3, 5, 4’) [3].

Compound **9**, Tetra-methoxypiceatannol.^1^H-NMR (500 MHz, CDCl_3_): 7.09 (3H, m, H-2’, 6’, 8), 6.94 (1H, d, *J* = 16.2 Hz, H-7), 6.89 (1H, d, *J* = 8.2 Hz, H-5’), 6.69 (2H, d, *J* = 2.2 Hz, H-2, 6), 6.41 (1H, t, *J* = 4.4, 2.2 Hz, H-4), 3.97 (3H, s, H_MeO_-3’), 3.92 (3H, s, H_MeO_-4’),3.85 (6H, s, H_MeO_-3, 5) [3].

The ^13^C NMR chemical shifts of compounds (**4**-**9**) were shown in **Table S1**.

**References**

1. Rueda DC, Schoffmann A, De Mieri M, Raith M, Jahne EA, Hering S, Hamburger M. Identification of dihydrostilbenes in Pholidota chinensis as a new scaffold for GABAA receptor modulators. Bioorg Med Chem. 2014;22(4),1276-1284.
2. Likhitwitayawuid K, Sornsute A, Sritularak B, Ploypradith P. Chemical transformations of oxyresveratrol (trans-2,4,3',5'-tetrahydroxystilbene) into a potent tyrosinase inhibitor and a strong cytotoxic agent. Bioorg med chem lett. 2006;16(21),5650-5653.
3. Adesanya SA, Ogundana SK, Roberts MF. Dihydrostilbene phytoalexins from Dioscorea bulbifera and D. dumentorum. Phytochemistry. 1989;28(3),773-774.
4. Jo G, Hyun J, Hwang D, Lee YH, Koh D, Lim Y. Complete NMR data of methoxylated cis- and trans-stilbenes as well as 1,2-diphenylethanes. Magn Reson Chem. 2011;49(6),374-377.

Table S1. ^13^C-NMR (125 MHz) spectroscopic data of compounds **4**–**9** (ppm)

|  | 4^a^ | 5^a^ | 6^a^ | 7^b^ | 8^b^ | 9^b^ |
| --- | --- | --- | --- | --- | --- | --- |
| 1 | 144.31 | 146.54 | 142.78 | 139.77 | 140.45 | 139.61 |
| 2 | 106.77 | 107.42 | 106.77 | 104.42 | 104.43 | 104.40 |
| 3 | 157.82 | 159.14 | 157.79 | 161.03 | 160.96 | 161.03 |
| 3-OMe |  |  |  | 55.40 | 55.40 | 55.39 |
| 4 | 99.77 | 101.08 | 99.78 | 99.68 | 98.45 | 99.76 |
| 5 | 157.82 | 159.14 | 157.79 | 161.03 | 160.96 | 161.03 |
| 5-OMe |  |  |  | 55.40 | 55.40 | 55.39 |
| 6 | 106.77 | 107.42 | 106.77 | 104.42 | 104.43 | 104.40 |
| 7 | 36.62 | 37.80 | 38.07 | 127.87 | 127.42 | 129.04 |
| 8 | 38.15 | 32.94 | 36.84 | 126.62 | 127.02 | 126.83 |
| 1’ | 132.79 | 121.05 | 133.66 | 129.98 | 123.90 | 130.30 |
| 2’ | 129.01 | 157.29 | 114.91 | 128.80 | 158.16 | 108.88 |
| 2’-OMe |  |  |  |  | 55.55 |  |
| 3’ | 114.64 | 103.53 | 144.53 | 114.20 | 99.47 | 149.19 |
| 3’-OMe |  |  |  |  |  | 55.98 |
| 4’ | 154.88 | 156.97 | 144.38 | 159.46 | 160.69 | 149.10 |
| 4’-OMe |  |  |  | 55.36 | 55.44 | 55.92 |
| 5’ | 114.64 | 108.24 | 115.27 | 114.20 | 105.06 | 111.29 |
| 6’ | 129.01 | 131.59 | 119.45 | 128.80 | 119.36 | 120.06 |
| a : ^13^C-NMR at 125 MHz in CD_3_OD; b : ^13^C-NMR at 125 MHz in CDCl_3_ | | | | | | |


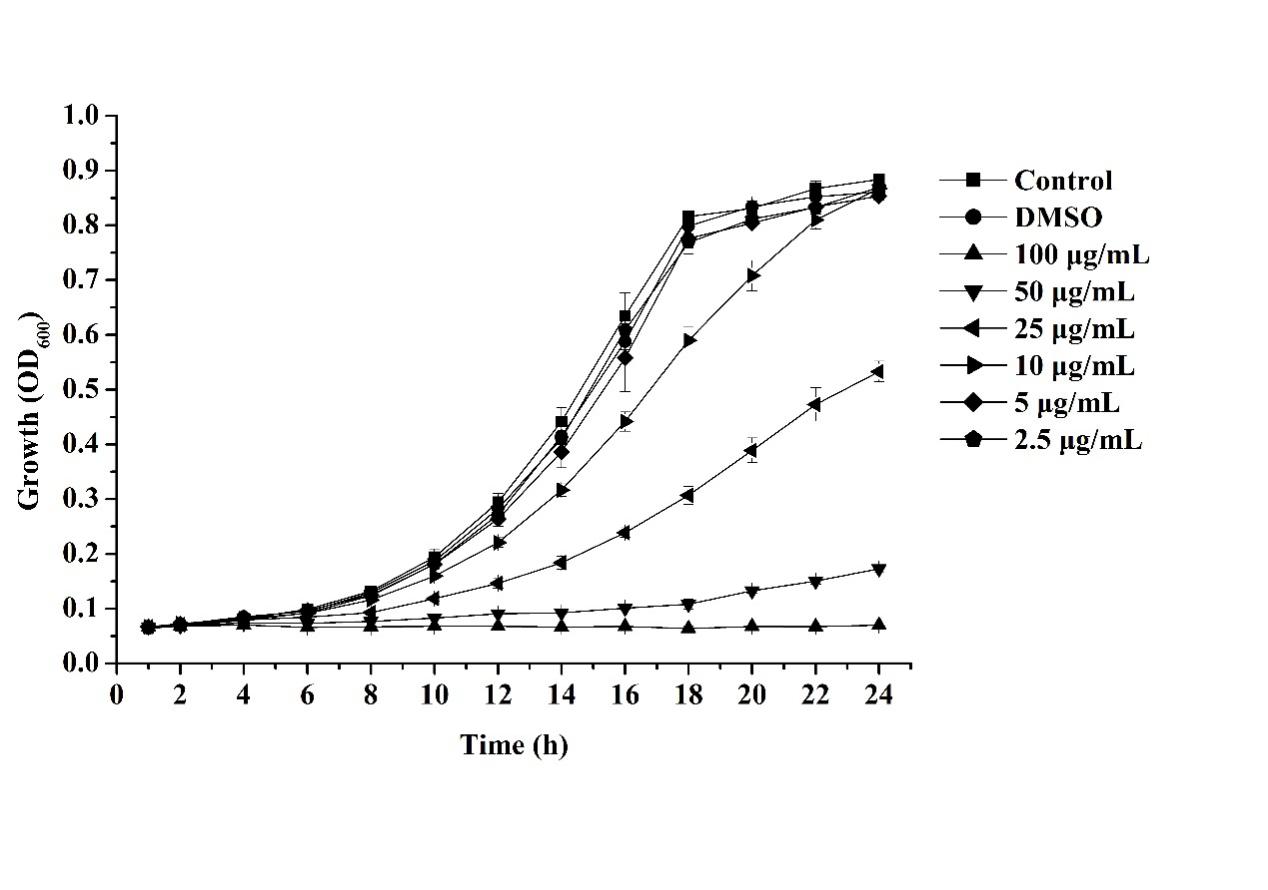


Fig. S1. Effects of resveratrol on *Xoo* growth with different concentrations.
